# Eccentricities in Diet

**Published:** 1889-06

**Authors:** 


### ECCENTRICITIES IN DIET.

"The preference of the Chinese for food that seems to our appetites absolutely disgusting, is well known. In Canton, rats sell for fifty cents a dozen, and dog's hind quarters command a higher price than lamb or mutton. Fancy eating birds' nests worth thirty dollars a pound! This is what a mandarin revels in. The French have beguiled us into eating frogs' legs, which were once tabooed in this country, and we have even come to esteem diseased goose liver, in the form of *pate de foie gras.* The writer has met Brazilians who rave over boa-constrictor steaks, and count monkeys and parrots a very good meal. In the West Indies, baked snake is a common dish, as the reptiles abound, and it is a good way of getting rid of them. But when it comes to frying palm worms in fat, one would think the stomach would rebel. It is not so, however, though, by a strange inconsistency, stewed rabbit is looked upon with disgust.

On the Pacific Coast the Digger Indians eat dried locusts, and in the Argentine Republic, skunk flesh is a dainty. Our own favorite bivalve, the oyster, is very disgusting to a Turk, while the devil fish, eaten in Corsica, is equally so to us. We cannot understand, either, how the inhabitants of the West Indies and the Pacific Coast can eat lizards' eggs with a relish ; still less, how the eggs of the turtle and alligator can become a favorite article of diet.

The Brazilians eat ants, probably, to get rid of them, for they literally infest the country, and are of an enormous size. It is easy to pick up a handful of ants almost anywhere, though the wary do not go about it in this way, as the petiferous insects bite in a most vicious manner. A curry of ants' eggs is a great delicacy in Siam, and the Cingalese eat the bees whose honey they have stolen. The Chinese, who seem to have stomachs like the ostrich, eat the chrysalis of the silk-worm, after unwinding the cocoon. Spiders are used in New Caledonia as a kind of dessert, while caterpillars are also relished by the African bushmen.

